# The association between a detectable HIV viral load and non-communicable diseases comorbidity in HIV positive adults on antiretroviral therapy in Western Cape, South Africa

**DOI:** 10.1186/s12879-019-3956-9

**Published:** 2019-04-27

**Authors:** S. George, N. McGrath, T. Oni

**Affiliations:** 1 Faculty of Medicine, University of Southampton, Southampton, UK; 2 Academic Unit of Primary Care and Population Sciences, Faculty of Medicine, University of Southampton, Southampton, UK; 3 Department of Social Statistics and Demography, Faculty of Social, Human and Mathematical Sciences, University of Southampton, Southampton, UK; 4 Africa Health Research Institute, KwaZulu-Natal, South Africa; 5 Division of Public Health Medicine, School of Public Health and Family Medicine, University of Cape Town, Cape Town, 7925 South Africa; 6 MRC Epidemiology Unit, Institute of Metabolic Science Building, Cambridge Biomedical Campus, University of Cambridge, Cambridge, CB2 0QQ UK

**Keywords:** HIV, Viral load control, Non-communicable disease, South Africa

## Abstract

**Background:**

Past studies have found a relationship between detectable HIV viral load and non-communicable diseases (NCDs) in HIV-infected individuals on antiretroviral therapy in high-income settings, however there is little research in South Africa. Our objective was to investigate the association between detectable HIV viral load and prevalent NCDs in a primary health centre in peri-urban South Africa.

**Methods:**

HIV-infected adults (aged ≥25) who had been on antiretroviral therapy for ≥ six months and attended the HIV clinic within a primary health centre in Khayelitsha, Cape Town, were recruited. We recorded participants’ demographics, HIV characteristics, the presence of NCDs via self-report, from clinic folders and from measurement of their blood pressure on the day of interview. We used logistic regression to estimate the association between a detectable HIV viral load and NCD comorbidity.

**Results:**

We recruited 330 adults. We found no association between a detectable HIV viral load and NCD comorbidity. Within our multivariable model, female gender (OR3·26; *p* = 0·02) age > 35 (OR 0·40; p = 0·02) low CD4 count (compared to CD4 < 300 (reference category): CD4:300–449 OR 0·28; CD4:450–599 OR 0·12, CD4:≥600 OR 0·12; p = < 0·001), and ever smoking (OR 3·95; p = < 0·001) were associated with a detectable HIV viral load. We found a lower prevalence of non-communicable disease in clinic folders than was self-reported. Furthermore the prevalence of hypertension measured on the day of interview was greater than that reported on self-report or in the clinic folders.

**Conclusions:**

The lack of association between detectable viral load and NCDs in this setting is consistent with previous investigation in South Africa but differs from studies in high-income countries. Lower NCD prevalence in clinic records than self-report and a higher level of hypertension on the day than self-reported or recorded in clinic folders suggest under-diagnosis of NCDs in this population. This potential under-detection of NCDs may differ from a high-income setting and have contributed to our finding of a null association. Our findings also highlight the importance of the integration of HIV and primary care systems to facilitate routine monitoring for non-communicable diseases in HIV-infected patients.

## Background

The rollout of antiretroviral therapy (ART) across Sub-Saharan Africa (SSA) has dramatically increased the life expectancy of those initiated on ART. As this population ages, they become increasingly susceptible to age-related comorbidity. An “accelerated ageing” syndrome has been described where deleterious features associated with ageing emerge decades earlier in HIV-infected individuals [1, 2]. Contributing factors are thought to be HIV itself and the effect of long term ART [2, 3].

To ensure optimal care for aging HIV-infected individuals, there is a need to recognize the association between comorbidity and HIV. Studies on ART patients have demonstrated the association between a detectable HIV viral load and a greater risk of developing comorbidities [4–6]. However, these studies were based in high-income settings where the HIV epidemic is not generalised. The rising prevalence of non-communicable diseases (NCDs) in SSA, combined with high levels of chronic infectious diseases such as HIV is resulting in a different pattern of multimorbidity than is seen in high-income countries [3]. Low-income groups in South Africa (SA) have seen a dramatic increase in prevalence of NCDs and have the highest burden of HIV, a chronic infectious disease resulting in concurrent epidemics [7]. The new ART initiation guidelines in SA will increase the numbers of patients on ART [8]. However, little is known about the impact of comorbid chronic diseases on long-term HIV management and care necessitating further research to better understand the association between NCD comorbidity and HIV control. In SA, NCD management is largely in primary care clinics, with treatment freely available. In the Western Cape province, primary care clinics are organised into disease-specific clubs, with a focus on four diseases (based on prevalence and importance as determined by contribution to disability adjusted life years, and the need for specialist expertise input in the primary care setting): diabetes, hypertension, asthma/chronic obstructive pulmonary disease, and epilepsy. The province-wide annual chronic disease audit conducted by the provincial government department of health, focuses on these diseases with the aim of improving management and disease outcomes. Yet, despite the increasing co-morbidity of NCDs with HIV in SA and an increasing body of research in high-income countries on the association between a detectable HIV load and NCDs, there is little known about the association between HIV and these diseases in low and middle-income settings [3–6]. The objective of this study was therefore to examine the association between prevalent NCDs of importance in this setting (hypertension, diabetes, epilepsy or chronic respiratory disease (CRD)) and a current detectable HIV viral load.

## Methods

The study was conducted in Ubuntu ART clinic, in Khayelitsha, the largest township on the outskirts of Cape Town, SA with approximately 500,000 predominantly black Africans and over 8000 HIV-infected individuals registered on ART [9, 10]. While Ubuntu clinic is located in a healthcare delivery complex that provides outpatient primary care services for NCDs, these facilities have separate buildings, staff and separate pharmacies; and represent non-integrated health systems. As a result, each HIV-infected patient at Ubuntu clinic has a clinic folder for ART only and (if diagnosis known) a separate folder for other primary health care services, including NCDs. There was no routine screening for NCDs in the ART clinic. Any recording of NCDs or attempt at screening was entirely based on the clinician’s decision at the time of consultation. As a result, there were no NCD data in many of the HIV clinic folders regardless of on-going care for NCDs in the primary healthcare clinic. The NCDs routinely managed in the primary care outpatient clinic and in routine NCD adherence clubs are diabetes, hypertension, CRD and epilepsy, in line with Western Cape Department of Health policy which annually audits these specific conditions [10].

Recruitment was from 13th January 2015 until 14th February 2015. During this period, as patients arrived at the clinic, staff transferred their HIV clinic folder to the clinic reception, where SG would screen the folders of the first ten patients for eligibility, and all those eligible were invited to participate. Once those eligible had participated, the next available ten folders were screened, this system was continued until the sample size was reached. Eligibility criteria were aged 25 years and older, on ART for at least 6 months and not knowingly pregnant at the time of interview. All participants gave written informed consent and interviews were conducted in a private clinic room on the same day. During the interview, data on the patient’s age, sex, current and past smoking habit, self-reported previously diagnosed comorbidities: hypertension, diabetes, chronic respiratory disease (CRD), and epilepsy were collected through administration of a questionnaire. These NCDs were selected as a priority due to their high prevalence in this population. In addition, these conditions represent health system priorities for the Western Cape province, as evidenced by the fact that NCD weekly outpatient clinics and adherence clubs exist for these conditions, and that the Department of Health annual audit for NCDs focuses on these conditions as a part of primary care service provision [4, 6, 10, 11]. The diagnosis of these NCDs is in line with guidelines set out by the South African government for primary care [12]. Specifically hypertension was diagnosed based on two elevated (> 140/90) blood pressure readings, diabetes diagnosed on the basis of a random blood glucose measurement > 11 and fasting blood glucose > 7, CRD was a clinical diagnosis based on symptoms and risk factors as set out in the primary care guidelines [12], and epilepsy diagnosed by a physician with specialist expertise based on the presence of two seizures with no other clear cause.

Including diabetes and hypertension enabled comparisons to be made with multimorbidity studies conducted in this setting [2]. Measurements (height, weight, and blood pressure using an electronic blood pressure machine) were taken once at the end of the interview by SG. The same technique and electronic blood pressure machine was used throughout the data collection period. After interview, the following data were extracted from the patient’s clinic folder and any missing data was extracted from the national health electronic laboratory service database: most recent viral load and CD4 count and date of ART initiation. This electronic database is an established national centralised service for the public healthcare system, with standard operating procedures for all tests, within which each patient, irrespective of what clinic they are seen, is identified via a unique identifier [13]. The primary outcome was a detectable viral load at the most recent viral load measurement, defined as > 40 copies/ml. We calculated that a sample size of 330 participants was sufficient to detect a two-fold higher proportion of patients with a detectable viral load among those with comorbidity compared to those with no comorbidity, with a power of 0·8 and an alpha level of 0·05, while assuming that half of the HIV population in the clinic would have at least one comorbidity listed above.

Four indicators of comorbidity (hypertension, diabetes, CRD and/or epilepsy) were created: i) comorbidity reported in clinic folder; ii) self-reported comorbidity during interview; iii) comorbidity reported in clinic folder OR on self-report during interview, and iv) a composite indicator consisting of comorbidity indicator (iii) (comorbidity reported in clinic folder OR self-report during interview) plus measured hypertension (diastolic ≥90 mmHg or a systolic ≥140 mmHg in line with SAGE, NIDS and SANHANES-1 studies [14, 15]) on the day of interview. These definitions of comorbidity are referred to throughout the rest of the manuscript as comorbidity indicators i, ii, iii, and iv. Comorbidity indicator iii was also used to generate a variable representing the number of diagnosed comorbidities recorded for each individual. Body mass index (BMI) was calculated using the height and weight data and categorised according to the WHO classification [16].

After excluding participants without a recorded viral load, the data were summarised descriptively, overall, and by gender. Characteristics were compared by gender using ***χ***^**2**^tests for categorical variables and Wilcoxon rank sum tests for continuous variables. Logistic regression models were used to examine the association between a detectable viral load and comorbidity, the number of comorbidities, and the four comorbidity indicators described above. In the multivariable model exploring the association between a detectable viral load and comorbidity, indicator iv) was used as the measure of overall comorbidity as it incorporated those who were measured as hypertensive on the day of recruitment into the study, comorbidity recorded in clinic folders and self-reported comorbidity.

Potential confounders included in the multivariable analysis were: smoking, BMI, CD4 count, age, and sex. Initially, smoking history was included in models using two indicators to represent never smoked, ex-smoker, and current smoker. BMI was considered using four indicators to represent underweight (BMI < 18·5), normal (BMI; 18·5–24·9), overweight (BMI 25–29·9) and obese (BMI ≥30).

However, in univariable analysis, odds ratio (OR) estimates were similar for the “ever smoked” and “current smoker” categories, and the normal and underweight BMI categories, and so these categories were grouped for parsimony in all subsequent analyses. We considered all variables that were significant at univariable level (*p* value < 0·10) for the multivariable model. For the final multivariable model, we explored interactions between sex and smoking, and sex and BMI given the known gender differences in smoking and BMI patterns in this population. All analyses were conducted using STATA SE 13 statistical package. Sensitivity analyses were also performed using data only for participants with a viral load measurement within 12 months of the interview date and re-running the final multivariable model.

## Results

**Fig. 1:**
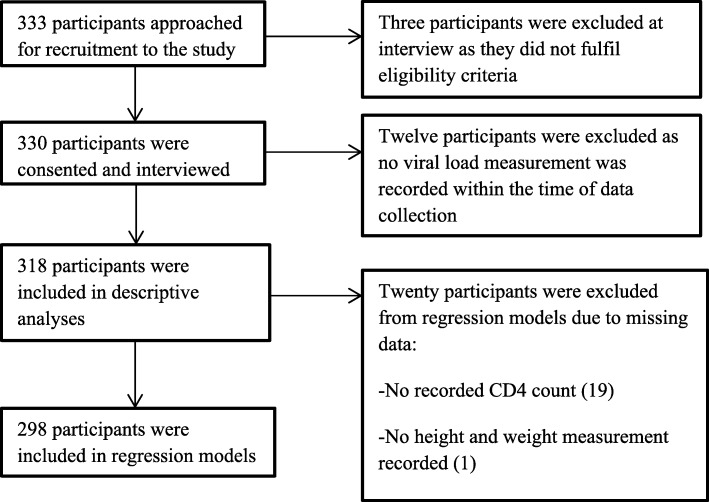
Flowchart of Participant Recruitment and Retention for Analysis

Figure 1 reports participant recruitment and retention for analysis. Among those who were eligible for analysis (*N* = 318, Table 1), 75% were female and the median age was 39 years among females and 42 years among males, *p* < 0·001. The median BMI for females and males was 29 and 22 (p = < 0·001) respectively, with 10% of males classified as obese compared to 45% of females. Significantly more men had ever smoked and were current smokers compared to women, 64% versus 9% ever smoked respectively (p = < 0·001), and 50% versus 4% were current smokers.

**Table 1:** Demographics of the sample, overall and by sex

Demographic characteristics	Sample Population *n* = 318 (%)	Male *n* = 80 (%)	Female *n* = 238(%)	Chi^2^ P Value
Age: Median (IQR)	39 (34–45)	*42 (37–47)*	*39 (33–44)*	*< 0.001* ^*c*^
*25–34*	82 (26)	14 (18)	68 (28)	0.07
*35–44*	145 (46)	35 (43)	110 (46)	
*45–54*	65 (20)	23 (29)	42 (18)	
*54 >*	26 (8)	8 (10)	18 (8)	
BMI: (*n* = 316; inc. 79 males)	28 (23–32)	*22 (19–26)*	*29 (25–34)*	*< 0.001* ^*c*^
Median (IQR)				
*Underweight*	20 (6)	*12 (15)*	*8 (3)*	*< 0.001*
*Normal*	96 (30)	*43 (55)*	*53 (22)*	
*Overweight*	85 (28)	*16 (20)*	*69 (30)*	
*Obese*	115 (36)	*8 (10)*	*107 (45)*	
Smoker				
*Yes currently*	50 (16)	*40 (50)*	*10 (4)*	*< 0.001*
*Ex-smoker*	23 (7)	*11 (14)*	*12 (5)*	
*Never*	245 (77)	*29 (36)*	*216 (91)*	
*Pack years amongst ever smokers: median (IQR)*	3 (1–6)	*4 (1–7)*	*1 (0–2)*	*< 0.001* ^*c*^

Significant results in italics ^c^ Wilcoxon rank sum test. N = 318 unless otherwise indicated

The median duration on ART was 4 years (IQR 2–7 years) and a significantly greater proportion of men had a low CD4 count (< 350) compared to women (46% vs 25% respectively; p = < 0·001) (Table 2). The median time since last CD4 measure was 8 months (IQR 4–12 months). Nineteen percent of the study population had a detectable HIV viral load (> 40 copies/ml), and 8% had viral load > 1000 copies/ml. The median time since last viral load was 7 months (IQR 4–11 months).

**Table 2:** Treatment-related characteristics, overall and by sex

Treatment-related Characteristics	Sample n = 318 (%)	Male n = 80 (%)	Female n = 238 (%)	Chi^2^ P Value
Duration of ART (years): Median (IQR)	4 (2–7)	3 (2–6)	4 (2–7)	0.09^c^
*0.5–2.5*	89 (27)	26 (33)	63 (26)	
*2.6–4.0*	76 (24)	25 (31)	51 (22)	0.09
*4.1–6.0*	59 (19)	12 (15)	47 (20)	
*> 6.0*	94 (30)	17 (21)	77 (32)	
Most recent Viral load (copies/ml)				
*Detectable*	60 (19)	18 (22)	42 (18)	0.34
*Not detectable (LDL/< 40)*	258 (81)	62 (78)	196 (82)	
Time since last VL: Median (IQR) (months)	*7 (4–11)*	5 (3–10)	7 (4–11)	0.21^c^
Most recent CD4 count (*n* = 299; inc. 76 males)				
Binary Indicator (cells/mm^3^):				
*Low (≤350)*	92 (31)	*35 (46)*	*57 (25)*	*< 0.001*
*High (> 350)*	207 (69)	*41 (54)*	*166 (75)*	
*Categorised CD4*				
*Low: 0–299*	73 (24)	*28 (37)*	*45 (20)*	*< 0.001*
*300–449*	58 (20)	*17 (22)*	*41 (19)*	
*450–599*	79 (26)	*21 (28)*	*58 (26)*	
*High: ≥600*	89 (30)	*10 (13)*	*79 (35)*	
Time since last CD4 measurement: Median (IQR) (months) (n = 299; inc. 76 males)	8 (4–12)	6 (3–11)	8 (4–12)	0.31^c^

Significant results in italics. ^c^ Wilcoxon rank sum test. N = 318 unless otherwise indicated

**Fig. 2:**
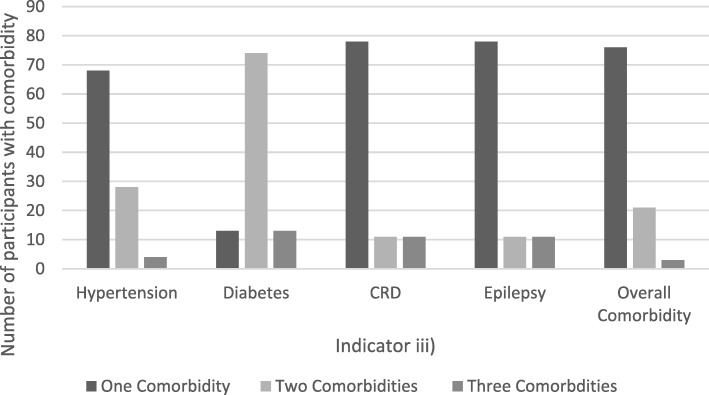
Number of participants with indicator iii) stratified by the number of comorbidities. CRD (Chronic Respiratory Disease)

**Fig. 3:**
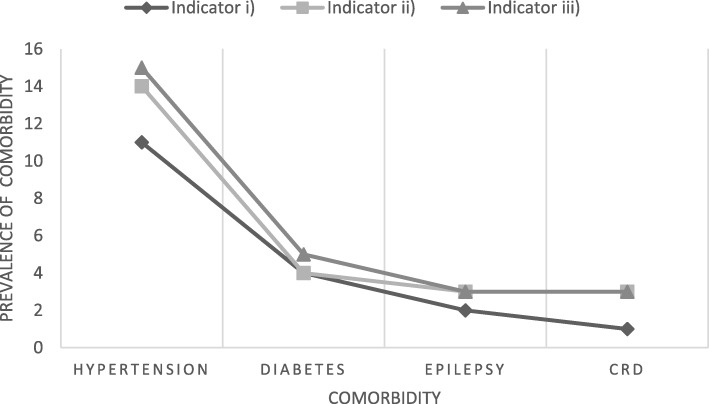
Prevalence of each comorbidity by indicators i) to iii). CRD (Chronic Respiratory Disease)

Overall the majority of participants had only one comorbidity (Fig. 2). Figure 3 illustrates the reported prevalence of comorbidities according to the source of diagnoses. Using indicator iii) (comorbidity on self-report or folder-review), the prevalence of comorbidity was 20% (95% CI 0.15–0.25), which was higher compared to using indicator i) (14% (95% CI 0.10–0.18)) or ii) (19% (95% CI 0.14–0.23)). While there was overlap between the two sources, additional reports came from each source individually. Overall, 69% of indicator ii) diagnoses had also been detected by indicator i), with 70, 92, 13, 57% of hypertension, diabetes, CRD, and epilepsy indicator ii) diagnoses also detected in indicator i) respectively. Of the 307 participants who had blood pressure measured on the day of interview (11 participants did not have blood pressure measured due to unavailability of an appropriate cuff size), 36% (*n* = 109) were found to be hypertensive. Of these, less than a third (28% (*n* = 31; 95% CI 0.21–0.38) had a previous diagnosis of hypertension. Including this diagnosis of hypertension on the day of interview in the definition of comorbidity (indicator iv)) increased the overall prevalence of comorbidities to 43% (95% CI 0.38–0.48).

In univariable regression models, none of the four comorbidity indicators, or the ordinal variable measuring number of comorbidities were significantly associated with a detectable HIV viral load (Table 3). In contrast, ever smoking, being normal/underweight (BMI ≤ 24·9), and having a low CD4 count (CD4 < 300 cells/mm^3^) were associated with higher odds of having a detectable viral load. The final multivariable model included age, sex, having ever smoked, BMI, CD4 count, and comorbidity indicator (iv) (Table 3). Significantly higher odds of a detectable HIV viral load were associated with being female (OR 3·26; *p* = 0·02), and having ever smoked compared to never smoked (OR 3.95; p = 0· < 001). Individuals aged ≥35 years had significantly lower odds of having a detectable HIV viral load compared to those aged < 35 years (OR 0·40; p = 0·02). Higher CD4 counts were also associated with significantly lower odds of a detectable viral load: compared to a referent of CD4 < 300 cells/mm^3^, CD4 300–449 cells/mm^3^ (OR 0·28; p = < 0·001), CD4 450–599 cells/mm^3^ (OR 0·12; p = < 0·001) and, CD4 ≥ 600 cells/mm^3^ (OR 0·12; p = < 0·001). BMI remained significant at the 10% level (*p* < 0·1) in the final model but effect estimates remained similar to the size of those in the univariate model suggesting normal / underweight was associated with higher odds of a detectable HIV viral load compared to being obese. The interactions between sex and smoking, and sex and BMI were in turn added to the final model, however neither interaction made a statistically significant contribution to the model and they were not retained. Sensitivity analyses were conducted excluding 52 individuals whose most recent viral load measurement was not within 12 months of interview. Excluding these individuals did not significantly change the model results (data not shown).

**Table 3:** Univariable and multivariable model of association with a detectable viral load

Variables	N of each variable. *N* = 298. (% that had a detectable viral load)	Univariable logistic regression: Odds ratio (Confidence Interval)	Likelihood Ratio Test P value for univariable model	Multivariable Odds ratio (Confidence Interval)	Likelihood Ratio Test *P* value for multivariable model
Age					*0.02*
25–34	75 (25)	1.00	0.08	*1.00*	
≥ 35	223 (16)	0.57 (0.30–1.07)		*0.40 (0.19–0.84)*	
Sex					
Male	76 (22)	1.00	0.32	*1.00*	*0.02*
Female	222 (17)	0.72 (0.38–1.36)		*3.26 (1.17–9.04)*	
Smoking					
Never smoked	228 (14)	*1.00*	*< 0.001*	*1.00*	*< 0.001*
Ever smoked	70 (34)	*3.32 (1,78–6.18)*		*3.95 (1.53–10.18)*	
BMI					
Obese	109 (12)	*1.00*	*0.01*	1.00	0.06
Overweight	82 (15)	*1.27 (0.54–2.94)*		1.15 (0.45–2.90)	
Normal/Underweight	107 (28)	*2.88 (1.40–5.89)*		2.57 (1.08–6.13)	
CD4					
Low: 0–299	73 (41)	*1.00*	*< 0.001*	*1.00*	*< 0.001*
300–449	58 (19)	*0.34 (0.15–0.75)*		*0.28 (0.12–0.67)*	
450–599	79 (9)	*0.14 (0.06–0.34)*		*0.12 (0.05–0.33)*	
High: ≥600	88 (8)	*0.12 (0.05–0.31)*		*0.12 (0.05–0.32)*	
Presence of any comorbidity (indicator iv)					0.15
No	168 (18)	1.00	0.76	1.00	
Yes	130 (19)	1.09 (0.61–1.97)		1.64 (0.84–3.23)	
Duration of ART					
0.5–2.5	81 (20)	1.00	0.24	–	–
2.6–4.0	73 (15)	0.72 (0.31–1.67)			
4.1–6.0	55 (27)	1.52 (0.68–3.41)			
> 6.0	89 (15)	0.70 (0.31–1.55)			
Previous diagnosis of comorbidity from clinic folders (indicator i)					
No	256 (18)	1.00	0.92	–	–
Yes	42 (20)	1.05 (0.46–2.41)			
Previous diagnosis of comorbidity from interview (indicator ii)					
No	244 (19)	1.00	0.44	–	–
Yes	54 (15)	0.73 (0.32–1.65)			
Previous diagnosis of comorbidity from either clinic folder or interview (indicator iii)					
No	240 (19)	1.00	0.51	–	–
Yes	58 (15)	0.77 (0.36–1.69)			
Diagnosis of hypertension on the day (*N* = 288)					
Not Hypertensive	186 (17)	1.00	0.49	–	–
Hypertensive	102 (21)	1.25 (0.68–2.30)			
Number of comorbidities					
0	240 (19)	1.00	0.45		
1	44 (16)	0.79 (0.33–1.90)			
≥ 2	14 (14)	0.70 (0.15 3.24)			

Significant results in italics

## Discussion

This study investigated the association between a detectable HIV viral load and the presence of comorbidities.

We found no statistically significant association between a detectable HIV viral load and the presence of any comorbidity (comorbidity indicator iv). This is consistent with a study conducted between 2004 and 2009 which found no association between a detectable HIV viral load and hypertension in HIV-infected adults on ART in SA [11]. In contrast, 2 studies based in the US over a similar time period examined HIV-infected adults on ART and found an association between comorbidity and a detectable HIV viral load [4, 6]. Neither study described the period of time participants were on ART and in one study all participants were treated for diabetes, hypertension, or both [4, 6]. Notably these studies had different definitions for viraemia. Two recent papers noted an association between low level viremia (> 50–1000 copies/ml) and increased risk of virological failure [17, 18]. While 8 % of our sample had a viral load of greater than 1000 copies/ml, due to insufficient statistical power, we did not sub-categorise viral load, and this may have contributed to our differing results. A second possible explanation is selection bias. Given that non-attendance is associated with a detectable HIV viral load, and all participants in our study were attending their clinic appointment, this could be a source of bias [19] as those within the study may be more likely than the general population to be virologically suppressed.

The high proportion of participants with measured hypertension on the interview day without a previous diagnosis of hypertension also indicates under diagnosis of comorbidity, and suggests that data reporting hypertension diagnoses are likely to be an underestimate of true prevalence [20, 21]. A meta-analysis looking at the prevalence of hypertension in SSA found that of those with hypertension, only 7–56% were aware of the diagnosis prior to the study [22]. White coat hypertension may contribute to the higher prevalence of measured hypertension, although research suggests that the presence of white coat hypertension may be associated with sustained hypertension in the future [23]. The prevalence of hypertension was consistent with that found in HIV-infected adults who had been on ART for over 1 year in SA [24]. However the prevalences of diabetes, CRD, and epilepsy found in this study were lower in comparison to another study surveying the SA adult population presenting to primary health care in 2010 [21]. This may be due to the fact that the majority of participants in that study were HIV uninfected. A study on HIV-infected persons over 50 years old in rural South Africa reported better functional ability, quality of life and overall health state (measured using three instruments: disability index, quality of life and composite health score) than HIV-affected participants. This suggests that enhanced healthcare received as part of HIV care could benefit overall wellbeing of HIV-infected older people [25]. However, there are little data on NCDs in HIV-infected persons [21].

Our results indicate that age < 35 years, female gender, ever smoking, and low CD4 count were associated with a detectable HIV viral load. Whilst this study had 75% female participants, this gender ratio is typical for ART clinics in this [26] and other settings [2, 27]. In addition, in Khayelitsha, this gender ratio persists in the NCD clinics due to the overall predominance of females attending primary care services [2]. The significant gender differences seen in BMI, smoking, and CD4 (Tables 1 and 2) may play a role. However, this study was not powered to stratify analyses by gender.

Comparing the comorbidity measurement between the HIV clinic folders and self-report, a lower level of comorbidity was found in the HIV clinic folders than was self-reported on interview. Inaccuracy in self-report may be a factor. For example, self-reported diagnoses of CRD were very few; possibly due to low levels of awareness of the diagnosis, or about CRD symptoms. It may also be a reflection of the absence of screening for NCDs in the HIV-infected population. The lack of integration between HIV and NCD healthcare systems at the time of this study is also a significant barrier to this ascertainment of NCD co-morbidity in this population group. Past studies have recognised this likely under-diagnosis of NCD alongside the paucity of data on NCD in HIV-infected individuals in LMICs [14, 20, 21]. This is particularly relevant as research suggests that HIV-infected individuals have a greater burden of NCDs than non-infected adults of the same age [2, 5, 28]. ART may also contribute, as studies have found that HIV-infected groups on ART have a higher prevalence of hypertension compared to similar groups not on ART [2, 29]. Amongst HIV-infected individuals with comorbidity in Soweto, SA, research suggests that rather than seeing their conditions as separate biomedical entities, patients transmute their conditions into an overall perception of chronic suffering [7]. This contrasts with the separation of healthcare services into distinct biomedical categories. The implications of the fragmented vertical systems are seen at Ubuntu clinic: separate folders for HIV and other primary healthcare service means treatment-related decisions, diagnoses, and measures of HIV control from one clinic may not be communicated to and recorded by all. Strengthening of health systems through integration of care is therefore needed, for example through sharing of clinic folders and data information systems, as well as implementation of routine active screening for common NCDs in HIV-infected persons. These strategies would serve to provide improved holistic chronic care for these patients, as well as enabling future research on the interaction between these co-occurring diseases [2, 3, 7].

Examining risk factors for comorbidity, our results highlight the importance of smoking and BMI as significant risk factors for comorbidity. Higher levels of smoking in HIV-infected compared to non-infected populations have been reported and previous studies have found that both HIV infection and smoking independently impact T-cell function and together significantly worsen the immune profile [30, 31]. Persistent immune activation is associated with increased morbidity and mortality [31]. Our finding that smoking was significantly associated with a detectable HIV viral load was also reported in a study of HIV-infected persons in the low HIV-burden setting of the United States of America [32] and merits further investigation given evidence that suggests tobacco smoking increases the immune activation in HIV-infected adults and is a known risk factor for NCD [31].

Non-nucleoside reverse transcriptase inhibitors antiretrovirals are used in SA and their side effects of lipodystrophy and truncal obesity may increase the risk of hypertension and diabetes [2]. This is particularly important in women due to the gender discrepancy in the prevalence of obesity found in this study, and others in SA [2, 33]. Previous research has recommended the inclusion of data on NCD risk factors such as smoking status and BMI in clinical HIV databases to encourage routine monitoring and to inform clinical decision making such as choice of ART drug prescription [31, 34]. It should however be noted that even in a vertical system, the CD4 count and viral load, important markers of disease control, were not always recorded in HIV clinic folders of the participants in this study. Given that these markers are an important aspect of HIV care, interventions to integrate inclusion of data on comorbidity risk factors and coexisting NCD should aim to also improve disease monitoring for HIV to avoid treatment failure and drug resistance [35].

As life expectancy and quality of life among persons with HIV improves due to the ART roll out, NCD co-morbidity is expected to increase, necessitating better integration between these vertical health systems [2, 3]. HIV/NCD co-morbidity is associated with increasing complexity in the management of comorbid diseases due to disease-drug and drug-drug interactions [28]. These interactions make access by clinicians to comprehensive information about patients necessary to inform care and decision-making.

### Strengths and limitations

This study addresses the need to investigate HIV/NCD comorbidity and the association with poor HIV viral suppression. However, there were some limitations. Despite the fact that 80% of HIV-infected individuals live in SSA and evidence describes a growing NCD prevalence in these HIV-infected populations, [2, 36] there is sparse evidence in LMICs about the effect of HIV/NCD comorbidity on HIV control [4, 6]. Furthermore there are little data on the models of care needed and possible interactions between these colliding epidemics and research in LMICs [36].

Our final sample had a slightly lower prevalence of comorbidity than hypothesised (43% vs 50%) and was smaller than the target number (298 vs 330), which may have reduced our ability to observe a statistically significant association between presence of comorbidity and detectable viral load. Some reduction in our final sample for analysis was because we were unable to measure blood pressure in all participants due to a lack of a sufficiently large cuff size for the most obese participants. Given the known link between hypertension and obesity, a greater proportion of this group may have been hypertensive. Thus, our estimate of hypertension prevalence may underrepresent the true level in this population. Furthermore, SA guidelines require multiple readings to make a diagnosis of hypertension. Although this was not feasible in this study, we did record previous diagnoses, in addition to measured elevated blood pressure. We did not measure adherence to medication. This may have been a confounder as past studies have suggested that those with poor adherence to ART, for a variety of reasons including substance abuse, a common risk factor in South Africa, may have poor adherence to medication for comorbidity resulting in a detectable viral load alongside higher blood pressure [6]. The gender ratio and sample size of this study limited the statistical power of this study to stratify the analyses by gender. Research suggesting that HIV-infected men have poorer health-seeking behaviour compared to women and hence a higher AIDS mortality rate highlight the importance of further research exploring the effect of gender on the association between viral load and multimorbidity; and the need for gender-specific strategies to encourage earlier enrolment into HIV care [27]. Given the vertical nature of the health system and absence of routine screening for NCDs in this HIV clinic, under-diagnosis of NCDs may have limited our ability to detect a significant association between NCD co-morbidity and viral load. Finally, the unknown timing of comorbidity diagnoses and the cross-sectional nature of our study meant we were unable to investigate causality in exploring comorbidity risk factor associations.

## Conclusion

We found no association between a detectable HIV viral load and comorbidity. Lower NCD prevalence in clinic records than self-report and a higher level of hypertension on the day than self-reported or recorded in clinic folders suggest under-diagnosis of NCDs in this population. This potential under-detection of NCDs may differ from a high-income setting and have contributed to our finding of a null association. This suggests the need for further research and better detection of, and screening for NCDs. An integrated chronic care system would allow enhanced detection, and dual management of HIV and NCDs and their risk factors, in addition to promoting an integrated approach to chronic infectious and NCD prevention.

## Abbreviations

ART: Antiretroviral Therapy
BMI: Body Mass Index
CRD: Chronic Respiratory Disease
LMIC: Lower/Middle-Income Country
NCD: Non-Communicable Disease
OR: Odds Ratio
SA: South Africa
SSA: Sub-Saharan Africa
